# Multiple Myeloma, Hyperviscosity, Hemodialysis Filter Clogging, and Antigen Excess Artifact: A Case Report

**DOI:** 10.1016/j.xkme.2021.02.011

**Published:** 2021-04-21

**Authors:** Swetha Rani Kanduri, Jason R. LeDoux, Karthik Kovvuru, Qingli Wu, Juan Carlos Velez

**Affiliations:** 1 Department of Nephrology, Ochsner Medical Center, New Orleans, LA; 2 Department of Pathology, Ochsner Medical Center, New Orleans, LA; 3 Ochsner Clinical School, The University of Queensland, Brisbane, Queensland, Australia

**Keywords:** Hook phenomenon, antigen excess, hyper viscosity syndrome, multiple myeloma, plasma exchange

## Abstract

Kidney involvement in multiple myeloma can result in kidney failure. Unlike Waldenström macroglobulinemia, hyperviscosity syndrome is a rare occurrence in multiple myeloma. Timely detection of hyperviscosity syndrome and initiation of plasma exchange to remove paraproteins can significantly alter the clinical course and be potentially lifesaving. We report a case of hospitalized patient with kidney failure due to multiple myeloma not in remission who experienced shortened hemodialysis sessions due to early dialysis filter failure due to hyperviscosity, which was later corrected with plasmapheresis. When confirmation of high levels of serum free light chains (sFLCs) was attempted, sFLC was initially reported as “not detectable.” This false-negative result reflected a laboratory artifact caused by a high abundance of sFLCs, known as antigen excess or hook phenomenon. Manual serial dilutions led to unmasking of markedly elevated κ light chain levels. This case exemplifies that patients with multiple myeloma can exhibit clinically challenging kidney manifestations even after becoming dialysis dependent.

## Introduction

Kidney involvement in patients with multiple myeloma is common.^1^ Hyperviscosity syndrome is a severe complication that necessitates emergent plasma exchange for paraprotein removal. Hyperviscosity syndrome, commonly described in Waldenström macroglobulinemia, is less frequently reported in patients with multiple myeloma (2%-6%).^2^ The revised International Myeloma Work Group has incorporated serum free light chain (sFLC) ratio as a biomarker in the diagnosis of multiple myeloma.^3^ Given their short half-life, sFLCs are clinically useful for monitoring response to therapy. However, sFLC assays could be subject to methodological variations, resulting in spuriously low values.^4^ We describe an unusual case of multiple myeloma in a patient with kidney failure dependent on hemodialysis presenting with hyperviscosity syndrome who later exhibited the antigen excess artifact.

## Case Report

A women in her mid-50s had multiple myeloma diagnosed 5 months before the index admission. She had an abnormal M band on the gamma portion of serum protein electrophoresis, immunofixation (IFE) gel suggestive of free κ light chain monoclonal protein, diffuse κ free light chains at 2,190 mg /dL on IFE, and κ:λ ratio of 3,696. A bone marrow biopsy specimen revealed 70% to 80% plasma cells and hypercellularity. She subsequently received 2 cycles of bortezomib, cyclophosphamide, and dexamethasone, and 1 cycle of high-dose cyclophosphamide, carfilzomib, and dexamethasone, the last cycle being 1 month before the index admission, without attaining clinical remission. Her kidney function declined and she required kidney replacement therapy. She was declared as having end-stage kidney disease 2 months before the index admission.

The authors were not directly involved in the care of the patient on the previous hospital admission. At that time, the treating physicians opted not to perform a kidney biopsy because it was believed that there was overwhelming clinical evidence to establish a clinical diagnosis of myeloma cast nephropathy (severe oliguric kidney failure and substantially elevated κ free light chains) and the risk for bleeding complications was deemed high (frailty and thrombocytopenia).

In the index admission, the patient presented to the emergency department with confusion and weakness. On examination, she was afebrile and tachycardic, with blood pressure of 150/90 mm Hg. Significant laboratory values included serum albumin level of 1.8 g/L, serum urea nitrogen level of 12 mg/dl, serum creatinine level of 5.1 mg/dL, κ light chain level < 0.04 mg/dL, and λ light chain level of 0.19 mg/dL. Computed tomography of the head and infectious workup were negative, and the patient did not report missing any of her dialysis sessions.

One hour into the patient’s scheduled hemodialysis session, the dialysis filter clotted and the treatment was interrupted. This session was resumed with the addition of a 2,000-unit heparin bolus and saline solution for prefilter dilution at blood flow rate of 250 mL/min, dialysate flow rate of 700 mL/min, and an F160 membrane (Fresenius Optiflux). However, the filter clotted again several times (once after 8 minutes and again after 15 minutes). Extremely elevated transmembrane pressure was recorded despite the administration of perceived adequate anticoagulation. Serum viscosity was then measured and found to be profoundly elevated at 7.88 centipoise (cP) (normal, 1.1-1.3 cP). A diagnosis of hyperviscosity syndrome was made and emergent plasma exchange was initiated.

At that time, serum protein electrophoresis and IFE reported elevated free κ light chains at 6,380 mg /dL; this contrasted with concurrent sFLCs analyzed by @Freelite SPA PLUS (The Binding Site Group Limited Birmingham) that revealed κ light chains < 0.04 mg/dL and λ of 0.19 mg/dL, values that did not correlate with other laboratory results or disease severity. This raised suspicion of the antigen excess artifact, or hook phenomenon. We conjointly worked with the clinical laboratory team and performed manual dilutions of 1:1,000 and 1:10,000, which resulted in substantially elevated κ light chain levels at 129,910 mg/dL. Her neurologic condition improved moderately after 3 sessions of plasma exchange. Unfortunately, the patient could not tolerate further rounds of chemotherapy due to side effects and her disease progressed. Multidisciplinary team discussions were held and the patient opted for hospice care.

## Discussion

Our case illustrates 2 important and relatively underappreciated concepts in the management of multiple myeloma. First, the phenomenon of hyperviscosity syndrome. Lambda free light chains exist as dimers with molecular weight of 45 kDa, as compared with κ light chains which exist as monomers with molecular weight of 22.5 kDa. As glomerular filtration rate declines, kidney clearance of sFLCs decreases and therefore the potential for light chain accumulation is high, with subsequent risk for hyperviscosity syndrome. Additionally, light chain clearance through the reticuloendothelial system is unaffected by the molecular weight of the light chains, such that the serum half-lives of both λ and κ light chains become similar, leading to an increased median sFLC ratio among patients with kidney failure.^5^ Although hyperviscosity syndrome is occasionally reported among patients with multiple myeloma,^2^ the presentation of recurrent hemodialysis filter clots secondary to hyperviscosity in a dialysis patient with multiple myeloma makes our case unique.

**Figure 1:**
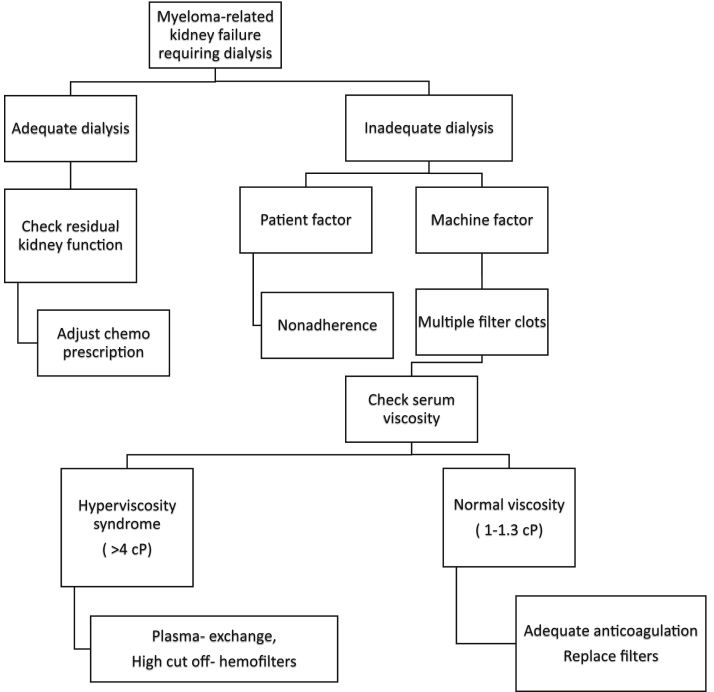
Algorithm for an approach to patients with multiple myeloma and kidney failure requiring kidney replacement therapy. Abbreviations: chemo, chemotherapy; cP, centipoise.

Timely initiation of plasma exchange to remove paraproteins can significantly alter the clinical course and be potentially lifesaving. The neurological condition of our patient improved moderately after 3 plasma exchange sessions. In addition to correcting hyperviscosity, which was the likely cause of clinical presentation, reduction in free light chain burden helped lower dialysis transmembrane pressures and allowed for successful completion of hemodialysis sessions. This case highlights the importance of considering plasma exchange in conjunction with hemodialysis in dialysis-dependent patients with multiple myeloma presenting with hyperviscosity (Fig 1).

The second concept encountered in this case was the co-existence of antigen excess, reported in 1% to 10% of samples from patients with multiple myeloma.^6^ This phenomenon is explained by a substantially elevated antigen concentration that exceeds antibody levels, resulting in spuriously low antigen antibody complexes and negative results. The hook phenomenon, as encountered with sFLCs, is synonymous to the prozone effect that is reported with intact immunoglobulins.^7^ Our patient had spuriously low reported κ and λ values. This finding likely was secondary to excess κ chain burden, overwhelming the concentration of antibodies and thereby impairing the assay performance. Polyclonal antibodies of the sFLC assay recognize epitopes on the constant region of light chains, which are exposed when light chains remain in free forms.^8^ Currently, multiple commercial assays are available for measuring sFLCs: Freelite,^9^ an immune nephelometric/turbidimetric assay; N latex FLC assay (Siemens Healthcare Diagnostics),^10^ based on monoclonal antibodies; and Sebia FLC, enzyme-linked immunosorbent assay (ELISA) based assays.^11^

Other methodological variations in analyzing sFLC assay include overestimation, which is secondary to polymerization of free light chains, exposing multiple epitopes and thereby generating falsely higher values.^12^ Nonlinear variations with a 2-fold or higher difference in sFLC values with various dilutions were reported as well.^13^ Lot-to-lot variation of sFLC concentrations of 8% to 45% have been reported with Freelite assay.^14^ Automated machine flags and delta checks can be incorporated to alarm unusual values.^15^ Sample dilutions,^4^^,^^16^ monoclonal antibodies,^17^ and more sophisticated modalities such as ELISA-based assays (Sebia FLC) and mass spectrometry help detect antigen excess.^11^^,^^18^ Table 1^4^^,^^6^^,^^11^, ^12^, ^13^, ^14^, ^15^, ^16^, ^17^, ^18^, ^19^, ^20^ describes the challenges in analyzing sFLC assays and potential recommendations.

**Table 1:** Methodological Variations in Analyzing Serum Free Light Chain Results and Recommendations

Methodological Variation in Analyzing Serum Free Light Chain Levels	Recommendations
Antigen excess/hook phenomenon^6^	Sample dilutions^4^^,^^16^ Delta checks^15^ Automated flags^15^ ELISA-based assay^11^ Mass spectrometry^18^ Monoclonal antibodies (less effect)^17^ Combination serologies, SPEP, UPEP, IFE^19^
Overestimation^12^	ELISA^11^ Mass spectrometry^18^
Non linearity^13^	Further dilutions^13^ ELISA, mass spectrometry^11^^,^^18^
Lot-to-lot variation^14^	Monoclonal antibodies (less effect)^17^
Cross-reactivity with intact immunoglobulin^13^	Monoclonal antibodies^20^

Abbreviations: ELISA, enzyme-linked immunosorbent assay; IFE, immunofixation; SPEP, serum protein electrophoresis; UPEP, urine protein electrophoresis.

In conclusion, prompt attention should be paid to dialysis-dependent patients with multiple myeloma who exhibit hemodialysis interruptions due to dialysis filter clotting, and have a low threshold to consider hyperviscosity syndrome. Early initiation of plasma exchange could be potentially lifesaving. Additionally, the antigen excess artifact, although rare is encountered in 1% to 10% cases of active multiple myeloma. ELISA-based assays and mass spectrometry are relatively more sophisticated and help mitigate some errors. Careful consideration of clinical context and combined interpretation of various serologic tests, including serum protein electrophoresis, IFE, and sFLC, is advised to guide clinical decision making.
